# Recent Patents of Interest to Dentists

**Published:** 1907-06

**Authors:** 


### PATENTS.

### RECENT PATENTS OF INTEREST TO DENTISTS.

851483. Moistening attachment for dental engines. George Bartlett, Lenapah, Indian, Ter.
851501. Tooth brush. Ernest L. Detrich, Cleveland, Ohio.
851550. Tooth brush guard. John C. Nevius, Trenton, N. J.
851578. Artificial teeth. Walter O. West, New Orleans, La.
852159. Dental tool handle. Joseph Bode, Philadelphia, Pa.
851735. Dental appliance. Chester M. Dowell, Elkhart, Ind.

852266. Artificial teeth. Ernest D. R. Garden, Tarrytown, N. Y.
852413. Removable dental bridgework. Ernest C. Bennett, New York, N. Y.
853031. Dental instrument. Augustus B. Prentis, Marshfield, Oregon.
853405. Tooth powder retainer, Ernest R. Godward, Invercargill, New Zealand.
853549. Tooth brush and powder receptacle. John P. Hill, Baltimore, Md.
853372. Machine for making wooden tooth picks. John H. Nute, Portland, Me.
853421. Dental chair. Frank Ritter, Rochester, N. Y.

Copies of above patents may be obtained for fifteen cents each by addressing John A. Saul, Solicitor of Patents, Fendall Building, Washington, D. C.

